# Functional Rhinoplasty

**DOI:** 10.7759/cureus.45993

**Published:** 2023-09-26

**Authors:** Alissa Altidor, Francisco A Ferri, Fadi Bakhos, Andres Mascaro-Pankova

**Affiliations:** 1 Medicine, Florida International University, Miami, USA; 2 Plastic and Reconstructive Surgery, Cleveland Clinic Florida, Weston, USA

**Keywords:** airway, breathing, nose, rhinoplasty, functional rhinoplasty

## Abstract

The nose is composed of intricate intranasal anatomy to serve its sophisticated functions. Although it only occupies a small area, it is the central focal point of the face and demands the highest level of understanding of the delicate interplay of form and function. Functional rhinoplasty, as opposed to aesthetic rhinoplasty, primarily aims to enhance nasal breathing and olfaction without altering the nose's appearance. The goal of this study is to describe the anatomy and physiology of the nose as well as the pathophysiology of nasal obstruction and the surgical approaches available for functional rhinoplasty. Whether when performed alone or combined with cosmetic rhinoplasty, functional rhinoplasty is a procedure that can bring significant benefits and improve the quality of life of our patients. Understanding nasal anatomy and physiology is key for successful management and outcomes. To provide optimal treatment for the patients, plastic surgeons must be familiarized with various techniques that have been documented.

## Introduction and background

Air enters the respiratory system through the nose, which is the only part of the system that is externally visible. The nose's key functions encompass warming, humidifying, and filtering the incoming air, which subsequently enters the lungs for gas exchange [1]. When these functions are impaired, nasal breathing can lead to substantial discomfort and a diminished quality of life [1]. Nasal obstruction has a range of anatomic and physiological factors, which compromises airflow. Common causes include septal deviations, stenosis, and turbinate hypertrophy [2]. Structural malfunctions of the nasal cavity can often be surgically corrected with functional rhinoplasty.

The nose is composed of intricate intranasal anatomy to serve its sophisticated functions. Although it only occupies a small area, it is the central focal point of the face and demands the highest level of understanding of the delicate interplay of form and function. The major components of the nasal valve contribute to normal nasal function. The septum, made up of quadrilateral cartilage, is the central structure of the nasal cavity that normally lies relatively straight down the midline to support the nose’s function and shape [3]. The inferior turbinates are lateral nasal wall structures made up of an independent bony structure that helps regulate nasal airflow. The internal nasal valve is formed by the septum, upper lateral cartilage, and inferior turbinate. This segment represents the most constricted section within the nasal airway, resulting in the highest overall impedance to airflow [4-6]. The initial point at which airflow resistance may manifest is within the external nasal valve [7].

Rhinoplasty is a surgical intervention that alters both the internal and external structure of the nose [8]. In contrast to cosmetic rhinoplasty, functional rhinoplasty primarily aims to enhance nasal breathing and the sense of smell without modifying the nose's appearance [8].

We believe functional rhinoplasty is a procedure that is not commonly performed by plastic surgeons. It can bring significant benefits and improve the quality of life of our patients, either when performed alone or combined with cosmetic rhinoplasty. The study aims to elucidate the nose anatomy, physiology, pathophysiology of nasal obstruction, and array of surgical approaches applicable to functional rhinoplasty.

## Review

Anatomy of the nasal cavity

Nasal Septum

The nasal septum separates the left and right nasal cavities [9]. The structure is composed of both bony and cartilaginous components [10]. The nasal septum is composed of the quadrangular cartilage anteriorly while in its posterior section, it is formed by the perpendicular plate of the ethmoid bone and the vomer bone [9]. The most inferior aspect of the septum is the nasal crest, which consists of the maxillary bone anteriorly and the palatine bone posteriorly [10]. The septum is also lined anteriorly by the mucoperichondrium (covering the quadrangular cartilage) and posteriorly by the mucoperiosteum (covering the bony septum) [11]. The nasal septum receives a well-developed blood supply, which stems from branches of both the maxillary and ophthalmic arteries [9]. The main function of the nasal septum is to provide support for the nose and regulate laminar airflow through the nasal cavities [1].

Inferior Turbinates

The inferior turbinates are a pair of structures composed of long, thin, curled bones that rest along the lateral nasal wall [3]. Superior to the inferior turbinates are the middle and superior turbinates, which both arise from the ethmoid bone. The inferior turbinates, however, are the largest turbinates and are separate bones themselves. The scroll-like, bony structures extend medially into the nasal cavity and contain an overlying mucosal layer made up of pseudostratified columnar respiratory epithelium [12]. The epithelium mainly consists of goblet cells, ciliated columnar cells, and basal cells [13]. Below the respiratory epithelium, there is a layer of erectile tissue comprising venous sinusoids responsible for draining the capillary network of the nasal mucosa [13]. The large surface area of the inferior nasal concha (turbinates) functions to filter, humidify, and warm inspired air and direct it toward the nasopharynx [1].

Internal Nasal Valves

The internal nasal valve stands as the most constricted segment within the nasal airway, making it the primary site for airflow resistance within the upper airway [3]. It is bordered medially by the cartilaginous septum, superolaterally by the caudal end of the upper lateral cartilages, anterolaterally by the head of the inferior turbinate, and inferiorly by the nasal floor [1]. The angle of the internal nasal valve should typically measure between 10 and 15 degrees in the nose of a Caucasian and is usually more obtuse in the African-American or Asian nose [12]. Attempts to enhance breathing within the internal nasal valve area typically involve the widening of this angle [3].

External Nasal Valves

Many define the external nasal valve as the opening of the nostril, but it is more clinically useful to regard the nostril opening as an element of the external nasal valve [6]. Positioned caudal to the internal nasal valve, the boundaries of the external nasal valve encompass the nostril opening caudally, the septum and medial crura medially, the alar cartilage and fibrofatty tissue in the anterolateral direction, and the opening of the internal nasal valve posteriorly [6]. This area serves as the entrance to the nose and represents the initial location where airflow resistance may arise [7]. The primary muscles responsible for maintaining the external nasal valve’s openness include the nasalis and dilator naris muscles [12].

Physiology of the nasal cavity

Normal airflow within the nasal vale region relies on the principles outlined by Bernoulli and Poiseuille [14]. The Bernoulli principle asserts that as airflow increases within a fixed space, the pressure within that space decreases correspondingly [14]. Poiseuille’s law, on the other hand, states that nasal resistance varies inversely with the fourth power of the radius of the nasal passages (resistance = (viscosity ∗ length)/radius^4) [12]. Poiseuille’s law serves as a tool for quantifying Bernoulli’s principle. Consequently, even minor adjustments in the radius of the nasal valves in either direction can have a significant impact on both airflow and resistance [6,12].

The nasal valve serves as the central location for controlling airflow and resistance, imparting the sensation of a properly functioning airway [12]. Inspiratory nasal airflow follows a parabolic curve through the nasal valve and optimizes contact between the airstream and the mucosal surface [15]. The physiology of the nasal valve resembles that of a Starling resistor [12]. During inspiration, a pressure gradient is developed between the nasopharynx and the atmosphere [6,12]. This is much like Starling, which stimulates changes in peripheral vascular resistance as the result of the change in the external pressure [6]. The narrow internal nasal valve works as a flow-limiting area that forcefully increases the speed and pressure of inspired air [16]. The internal and external nasal valves function together to deliver laminar airflow to the nasal cavities for humidification [15].

The respiratory region of the nasal cavity plays an important role in the transformation of the nasal airflow from a laminar pattern to a more turbulent pattern [16]. The deceleration of airflow along with increased turbulent forces contribute to humidification, heating, filtering, and olfaction [7,15].

Nasal obstruction

Nasal blockage is a frequently encountered symptom by both general practitioners and otolaryngologists and can result from a broad spectrum of anatomical, physiological, and pathophysiological factors [7]. Approximately one-third of the population reports nasal obstruction as a concern and turns to their healthcare providers for assistance [7].

Nasal obstruction results from a dynamic interaction of static and dynamic forces. Static forces are defined as stenotic areas. Examples of nasal obstruction secondary to static forces include septal deviation, turbinate hypertrophy, inherent concavity of the nasal cartilages, and avulsion of the attachment of the upper lateral cartilages to the septum [17]. Dynamic forces are defined as a lack of resiliency of the cartilages, leading to collapse. This is usually secondary to weakened cartilage or dilator muscles [17].

Patient evaluation

The subjective evaluation includes nasal-facial analysis during inspiration and expiration, palpation of the cartilaginous frame, intranasal examination, including anterior rhinoscopy and endoscopy, Cottle and modified Cottle maneuvers and quality of life measures such as nasal obstruction symptom evaluation (NOSE) and visual analog scale (VAS). The objective measurements of nasal obstruction include rhinomanometry, acoustic rhinometry, imaging studies, and rhinoresiliography.

Rhinomanometry stands as the most frequently employed objective assessment method for evaluating the nasal airway. Its primary objective is to gauge airflow in relation to air pressure individually for each nasal passage [17]. To assess one nasal passage in isolation, a pressure-sensing plug is used to block the opposite nostril, and the airflow through the unobstructed passage is measured using a flow sensor integrated into a snug-fitting facial mask [17].

Acoustic rhinometry determines the cross-sectional area of the nasal cavity in relation to the depth within the nasal passage, starting from the nasal sill [1]. This technique operates by examining alterations in acoustic reflections when sound waves traverse the nasal cavity, generating graphical data [1].

Computed tomography (CT) and magnetic resonance imaging (MRI) have been employed in radiographic evaluations of nasal blockage [1]. Both modalities facilitate the assessment of the nasal valve angle [1]. The nasal base view, conducted in a plane perpendicular to the anterior aspect of the estimated acoustic axis, is considered to closely approximate the measurement of the internal nasal valve [1].

The rhinoresiliography assesses the elasticity and bounce-back capability of the nasal tip concerning the applied force and distance [18]. A transducer is linked to the nasal tip, capable of gauging the force exerted on the tissue (resilience) and the force received from the tissue (recoil) [18]. The underlying principle is that the force required to displace the tissue is greater than the force generated by the tissue to return to its initial shape [18].

Surgical techniques

**Figure 1:**
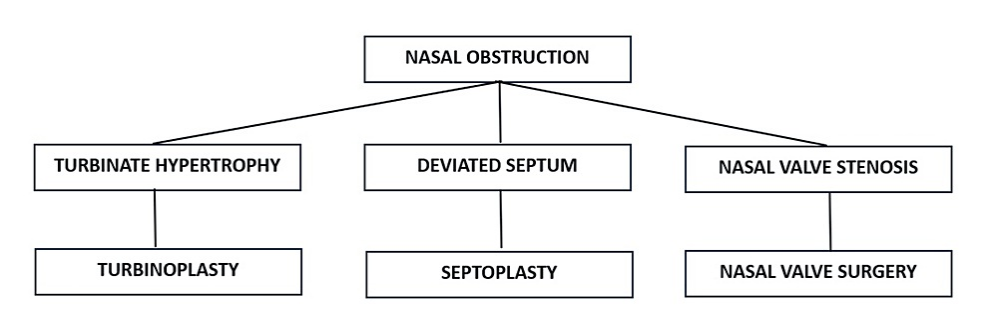
Surgical options to address nasal obstruction

The surgical management of nasal obstruction aims to address a deviated septum, turbinate hypertrophy, and/or nasal valve stenosis. This encompasses septoplasty, turbinoplasty, and nasal valve surgery (Figure 1). These procedures can be conducted either independently or in conjunction, with the primary objective of alleviating nasal obstruction and enhancing the patient's breathing.

Septoplasty

A straight alignment of the nasal septum along the midline is crucial for maintaining the nose form and function [3]. Any deviation or deformity in the nasal septum can lead to nasal obstruction, a prevalent concern in the realm of rhinology [19]. Septal deviations can result from trauma or congenital factors, and their nature and severity may vary depending on their location (e.g., caudal, posterior, anterosuperior) [3]. To address structural abnormalities, septoplasty is a common surgical procedure [19]. Surgeons can perform this procedure using either a headlight and nasal speculum or an endoscope [10].

The endoscopic approach offers enhanced visualization of tissue planes, along with improved illumination and magnification. This enables the surgeon to assess nasal anatomy with greater precision and selectively remove segments of the deviated septum [19]. This capability is particularly advantageous for cases involving isolated septal issues, especially those with more posterior deviations [19].

Complex cases, such as caudal deviations (crooked nose) or cases that may involve other structures, are best treated with an open rhinoplasty [20]. The open approach allows the surgeon to simultaneously contact the caudal and dorsal septum [20]. In order to prevent loss of nasal support after correcting the septal deformity, grafts of septal cartilage or ethmoidal bone may be sutured in place for reinforcement [5]. Unique challenges may arise with caudal septal deviations [10]. The condition tends to result in stenosis of the internal nasal valves and manipulation or removal of the caudal septal support risks creating nasal deformities [3,10]. Resection of the deviation must improve the nasal airway and reconstruction must be done in a way that does not jeopardize nasal tip support [10]. In many standard cases, a combined approach of endoscopic and open rhinoplasty may also be utilized to take advantage of the distinctive benefits provided by each approach [19].

Turbinoplasty

Chronic enlargement of the inferior turbinates can result in nasal airway blockage [3]. When topical anti-inflammatory treatments prove ineffective, various surgical approaches are contemplated for reducing turbinate size, including lateral out-fracture, submucosal diathermy, and partial or complete removal [13]. Turbinate surgery is only considered when it is absolutely necessary, as many patients experience satisfactory functional improvements through the correction of nasal valve and septal irregularities without the need for turbinate resection surgery [3]. The most frequent surgical method, turbinate reduction, can cause destruction to the respiratory mucosa. Therefore, this technique is ideally performed to preserve as much of the nasal mucosa as possible to maintain its capacity to secrete mucus and humidify the inhaled air [3,13].

Nasal Valve Surgery

**Figure 2:**
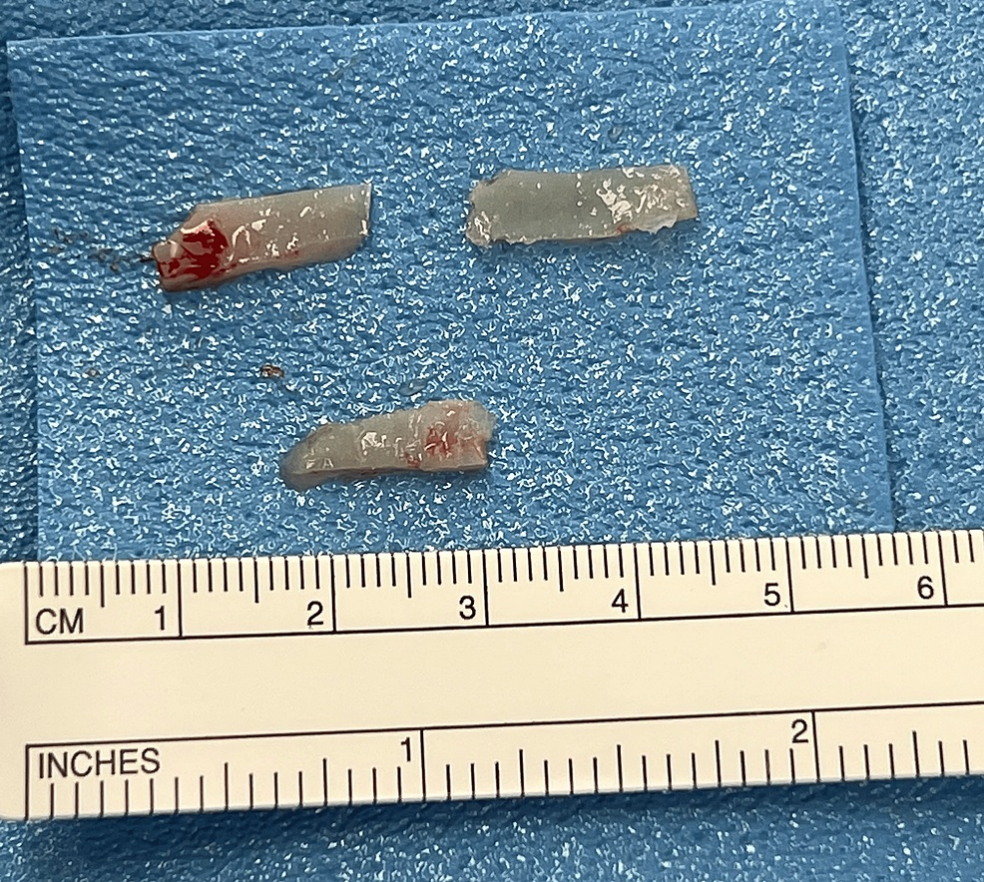
Cartilage graft harvested from the nasal septum

**Figure 3:**
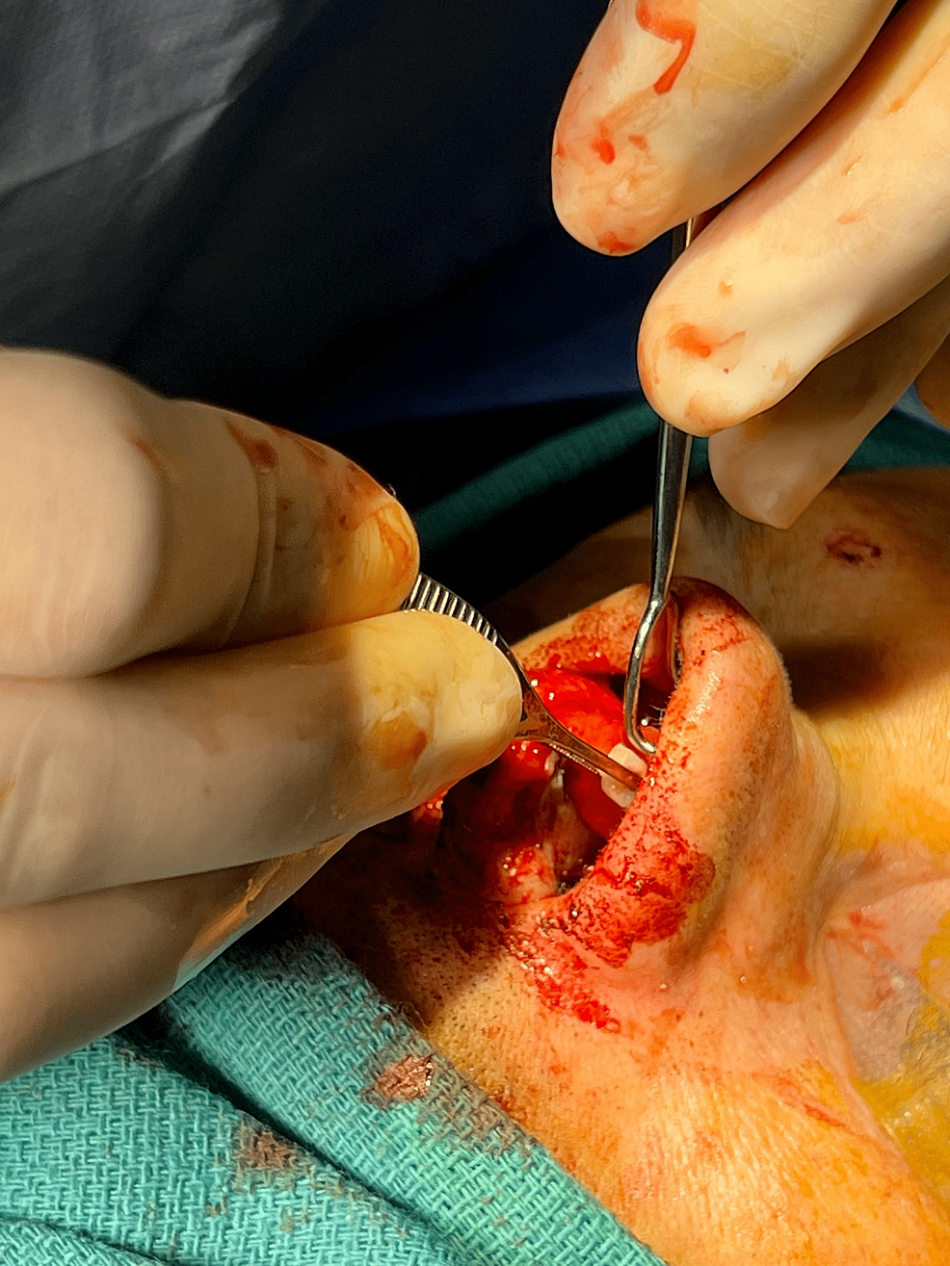
Placement of septal cartilage as spreader grafts between the upper lateral cartilages and the septum

**Figure 4:**
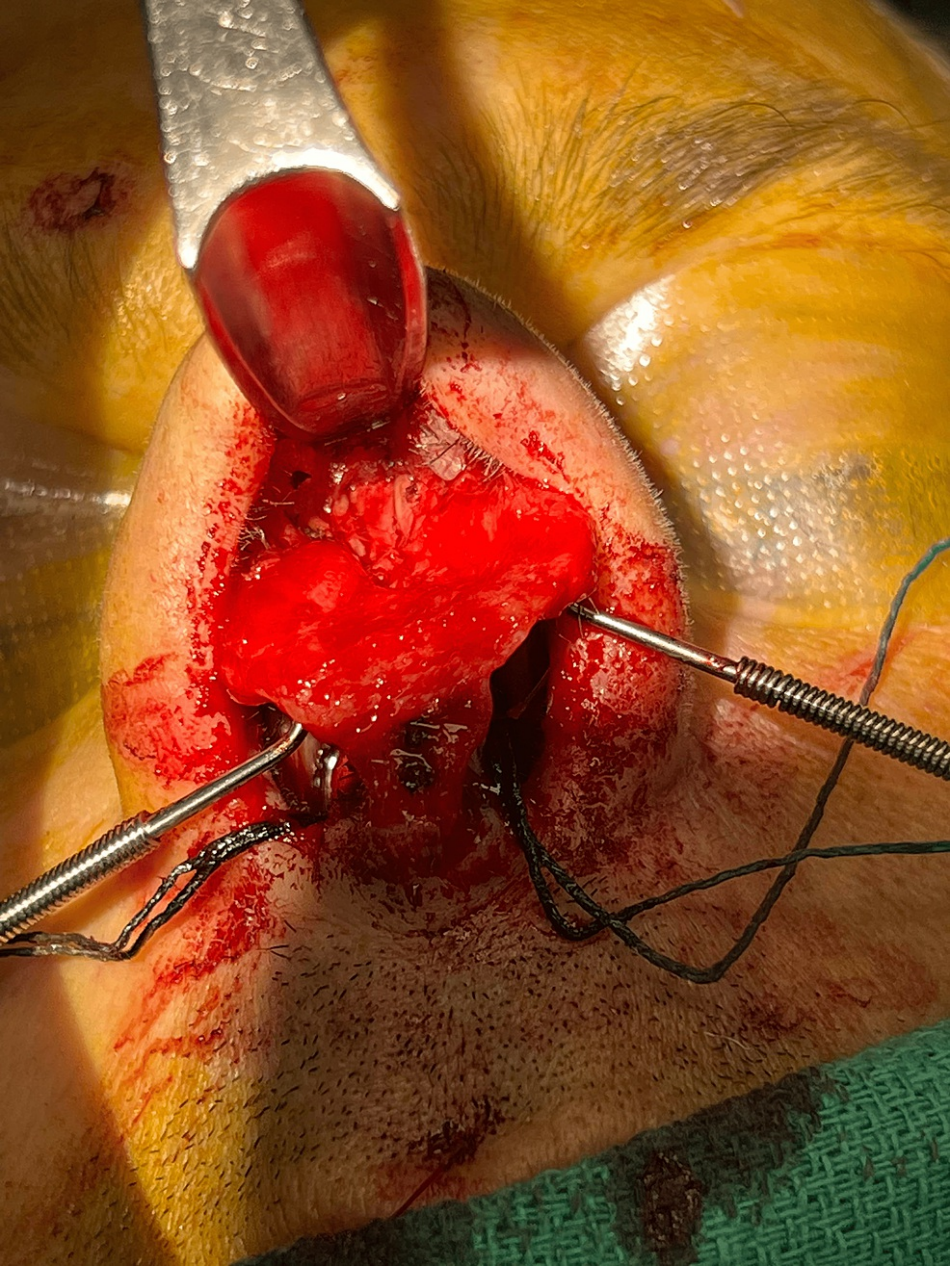
Final results after spreader grafts placement

Internal nasal valve abnormalities can be corrected with several surgical techniques that can be performed with an open or endonasal approach [12]. The commonly utilized approach for addressing this region involves inserting spreader grafts (as depicted in Figures 2-4) between the upper lateral cartilages and the septum [21]. Alternatively, the upper later cartilage can be used to create autospreader flaps. These grafts work to significantly increase the cross-sectional area of the internal nasal valve [3]. The flaring suture presents another technique employed to increase the angle and cross-sectional area of the internal nasal valve [14]. Vertical mattress sutures are positioned between the lower ends of the upper lateral cartilages and secured over the dorsal septum [21]. This suture influences both static constriction and dynamic collapse by widening the internal nasal valve and introducing tension to counteract sidewall collapse [14].

The butterfly graft is another highly effective method for addressing internal nasal valve blockage [3]. Obtained from the conchal cartilage of the ear, the graft is meticulously sculpted into the desired shape and placed above the anterior septal angle and the lower end of the upper lateral cartilage [14]. By inserting the lower part of the graft deeply into the uppermost region of the lower lateral cartilage, it additionally provides support to the external nasal valve, resulting in a dual effect that highlights the substantial influence of this technique on the nasal valve [14].

The mainstay of treatment of external valve collapse has been alar batten grafts [12]. These grafts are sourced from the septal or conchal cartilage and augment the weak or absent lower lateral cartilage along the nasal sidewall or alar rim [3]. Placed in line or caudal to the lateral crus, the graft is made thin and elastic to keep out of the airway and durable for maximum structural support [6]. Alternative to the alar batten grafts are the alar strut grafts that underlay the lateral crura instead of overlay [6]. The graft is positioned by lifting the vestibular skin on the underside of the lateral crus and is typically placed in a more caudal location compared to the structure itself [12]. This technique is a reliable way to flatten and straighten the lateral crus and provide support to the external nasal valve [6,12]. Alar rim grafts are effective in reinforcing the nostril margin [6]. The thin cartilage graft spans the alar rim region and improves the contour and strength of the rim [14]. The alar rim graft offers a practical remedy for addressing lower-third nasal deficiencies without causing substantial alterations to the overall appearance of the nose [3]. Tip ptosis correction and deprojection are other alternatives that will cause the nostrils to flare, increasing the area of the nostril opening. Caution must be taken since the cosmetic appearance of the nose may change.

## Conclusions

Functional rhinoplasty is a procedure that can bring significant benefits and improvement to the quality of life of our patients, either when performed alone or combined with cosmetic rhinoplasty. Understanding nasal anatomy and physiology is key for successful management and outcomes. Plastic surgeons must be familiarized with several distinct methods that have been described in order to offer the best possible treatment for the patient.

## References

[1] Sowder JC, Thomas AJ, Ward PD (2017). Essential anatomy and evaluation for functional rhinoplasty. Facial Plast Surg Clin North Am.

[2] Osborn JL, Sacks R (2013). Nasal obstruction. Am J Rhinol Allergy.

[3] Friedman O, Cekic E, Gunel C (2017). Functional rhinoplasty. Facial Plast Surg Clin North Am.

[4] Pawar SS, Garcia GJ, Rhee JS (2017). Essential anatomy and evaluation for functional rhinoplasty. Facial Plast Surg Clin North Am.

[5] Teichgraeber JF, Gruber RP, Tanna N (2016). Surgical management of nasal airway obstruction. Clin Plast Surg.

[6] Hamilton GS 3rd (2017). The external nasal valve. Facial Plast Surg Clin North Am.

[7] Hsu DW, Suh JD (2018). The external nasal valve. Otolaryngol Clin North Am.

[8] Oneal RM, Beil RJ (2010). Surgical anatomy of the nose. Clin Plast Surg.

[9] Kuan EC, Palmer JN (2021). Surgical anatomy of the nose, septum, and sinuses. Endoscopic Surgery of the Orbit.

[10] Most SP, Rudy SF (2017). Septoplasty: basic and advanced techniques. Facial Plast Surg Clin North Am.

[11] Smith DH, Brook CD, Virani S, Platt MP (2018). The inferior turbinate: an autonomic organ. Am J Otolaryngol.

[12] Lee J, White WM, Constantinides M (2009). Surgical and nonsurgical treatments of the nasal valves. Otolaryngol Clin North Am.

[13] Georgakopoulos B, Hohman MH, Le PH (2023). Anatomy, Head and Neck, Nasal Concha. https://www.ncbi.nlm.nih.gov/books/NBK546636/.

[14] Barrett DM, Casanueva FJ, Cook TA (2016). Management of the nasal valve. Facial Plast Surg Clin North Am.

[15] Manickavasagam J, Wong S, Varabei V, Raghavan U (2014). Nasal valve surgery: assessment of quality of life with the Glasgow Benefit Inventory. Ear Nose Throat J.

[16] Seren E, Seren S (2009). Morphological adaptation of the nasal valve area to climate. Med Hypotheses.

[17] Ghosh A, Friedman O (2016). Surgical treatment of nasal obstruction in rhinoplasty. Clin Plast Surg.

[18] Rhee JS, Poetker DM, Smith TL, Bustillo A, Burzynski M, Davis RE (2005). Nasal valve surgery improves disease-specific quality of life. Laryngoscope.

[19] Shah J, Roxbury CR, Sindwani R (2018). Techniques in septoplasty: traditional versus endoscopic approaches. Otolaryngol Clin North Am.

[20] Stevens MR, Emam HA (2012). Applied surgical anatomy of the nose. Oral Maxillofac Surg Clin North Am.

[21] Fattahi T (2008). Internal nasal valve: significance in nasal air flow. J Oral Maxillofac Surg.

